# Investigating residual leukemic cells in acute lymphoblastic leukemia: a practical approach using a streamlined interphase fluorescence in situ hybridization method on cerebrospinal fluid

**DOI:** 10.1186/s13039-023-00649-x

**Published:** 2023-07-27

**Authors:** Knarik Karapetyan, Mane Gizhlaryan, Olga Kalinovskaia, Anna Hovhannisyan, Gohar Tadevosyan, Lilit Matinyan, Gevorg Tamamyan, Narine Ghazaryan

**Affiliations:** 1 Department of Molecular Biology, Hematology Center After Prof. R.H.Yeolyan, Yerevan, Armenia; 2 Pediatric Cancer and Blood Disorders Center of Armenia, Hematology Center After Prof. R.H: Yeolyan, Yerevan, Armenia; 3 Department of Hematology, Yerevan State Medical University, Yerevan, Armenia; 4 Department of Pediatric Oncology and Hematology, Yerevan State Medical University, Yerevan, Armenia; 5 Laboratory of Toxinology and Molecular Systematics, Institute of Physiology, Yerevan, Armenia; 6 Department of Medical Genetics, Yerevan State Medical University, Yerevan, Armenia

## Abstract

**Introduction:**

A precise diagnosis of central nervous system involvement in acute lymphoblastic leukemia (ALL) requires comprehensive knowledge of morphological analysis, with a focus on the quantity and quality of cells being examined. Some research has utilized techniques such as immunocytochemistry, flow cytometry, polymerase chain reaction (PCR), and interphase fluorescence in situ hybridization (iFISH) on cerebrospinal fluid (CSF) cytospin samples to detect any remaining leukemic cells in the CSF. To obtain reliable results using immunocytochemistry and flow cytometry, it is essential to use freshly collected specimens within a limited timeframe. At the same time, PCR requires a sufficient number of cells for DNA extraction. On the other hand, the iFISH procedure on CSF cytospin samples can be challenging and requires practice. Therefore, there is a need for a fast, easy method that will be affordable and marketable in laboratories where the above methods are not available, or the sample is insufficient to use those methods.

**Methods:**

The samples were prepared by centrifugation of 1 mL aliquots of CSF collected into EDTA tubes. The CSF sample was centrifuged at 3000 rpm for 3 min, the supernatant was removed, and the pellet was placed in KCl hypotonic solution for 5 min at 37 °C. Other steps (fixation, hybridization, wash steps, and analysis) were the same as in the standard protocol for blood samples. The BCR-ABL1 rearrangements were performed and evaluated in 200 interphase cells.

**Results:**

90% of Ph(+) cells were found in CSF.

**Conclusion:**

We propose a significantly streamlined iFISH method for detecting blast/residual leukemic cells in acute lymphoblastic leukemia using CSF as a complementary test option.

## Introduction

Accurately detecting the central nervous system (CNS) involvement in children with acute lymphoblastic leukemia (ALL) is pivotal at the initial diagnosis and follow-up after treatment. Less than 2% of pediatric patients have CNS involvement at the initial diagnosis, and CNS relapses can be reduced to less than 5% after CNS prophylaxis [1, 2]. However, CNS involvement is still observed in more than 30% of all relapses in isolated CNS relapse or combined with bone marrow (BM) relapse. Leukemic blasts in cerebrospinal fluid (CSF) are associated with an increased risk for CNS relapse if adequate therapy is delayed [3].

The patients are considered at increased risk of CNS relapse if the detection of blast cells in CSF is accompanied by a CSF-WBC count exceeding 5 cells/μl. Based on this, a specific risk score was generated: CNS1, denoting the absence of identifiable leukemic cells in CSF; CNS2, indicating the presence of blast cells in a CSF sample containing < 5 WBC/μl; and CNS3, a CSF sample that has ≥ 5WBC/μl together with identifiable blast cells, or the presence of cerebral mass, or cranial nerve palsy together with leukemic cells in the CSF [4].

ALL is the most common childhood cancer. Approximately 25% of adults and 5% of children diagnosed with ALL have the Philadelphia chromosome, also known as the BCR-ABL1 rearrangement. This rearrangement involves the fusion of the BCR gene located on chromosome 22 with the ABL gene on chromosome 9, creating a constitutively active tyrosine kinase fusion protein. The presence of the Philadelphia chromosome in ALL is linked to a more severe form of the disease and is considered a poor prognostic factor, especially for children. As a result, patients with Ph + ALL are often treated with more aggressive methods, such as bone marrow transplantation. Despite these efforts, the relapse rate for Ph + ALL remains high, ranging from 40 to 80% [5–7].

The diagnosis of CNS involvement in ALL continues to pose a challenge due to underdiagnosis. The current standard diagnostic method is the morphological examination of CSF. Accurately identifying blast cells in CSF is crucial. Still, distinguishing between blast and healthy cells can be difficult, especially when the cell count is low [8]. Cytospin preparations of CSF are commonly used, but the interpretation accuracy depends on the examiner's experience and the specimen's quality [4, 9, 10].

While PCR and NGS have been suggested for detecting residual leukemic blasts in CSF, both require adequate cell numbers for nucleic acid extraction [8, 11–13]. Additionally, immunocytochemistry and flow cytometry are used in conjunction, but both require fresh samples and a sufficient number of cells [14–16]. Due to the limitations of these methods, there is a need for a faster, more straightforward, and more accessible way for laboratories where the techniques mentioned above are unavailable or samples are insufficient. We propose a standard interphase fluorescence in situ hybridization (iFISH) method of CSF as a supplementary test option for identifying the presence of blast cells or residual leukemia cells in ALL cases.

## Methods

### iFISH and slide preparation

We performed iFISH analyses on the CSF sample to identify the blast cells. iFISH on this sample was performed using a ZytoLight ® SPEC BCR/ABL1 dual color dual fusion probe (Germany) and evaluated 200 interphase cells. The samples were prepared by centrifugation of 1 mL aliquots of CSF collected into EDTA tubes. The CSF-WBC count was 1300 cells/μl in positive sample and 19 cells/μl in negative sample. After centrifugation at 3000 rpm for 3 min, the supernatant was removed, and the pellet was placed in KCl hypotonic solution for 5 min at 37 °C. The remaining steps, such as fixation, hybridization, washes, and slide preparation, followed the standard protocol for blood samples. For the iFISH procedure, the slides were soaked in 99% ethanol, rinsed with dH2O, aged at room temperature for 2 h, dehydrated in an ethanol series (75%, 85%, and 96%), and then the IVD BCR/ABL1 t(9;22) fusion FISH probe was applied. We added 6 μL of probe to each slide and covered it with a glass coverslip (22 × 22 mm). Denaturation was performed in ThermoBrite (Abbott Molecular) at 75 °C for 5 min, followed by overnight hybridization at 37 °C with humidity. Post-hybridization washes included 2xSSC at 75 °C for 3 min in a water bath, followed by 0.4xSSC/Tween 0.05% at room temperature. Subsequently, dehydratation in an ethanol series (75%, 85%, and 96%) was performed, and the nuclei were stained with DAPI [17]. The cutoff for our analyses is set at 3% which was calculated using the inverse beta distribution. In our study, we examined 20 healthy individuals as a control group, analyzing 200 cells for each analysis.

## Result

The male patient born on 02.08.2007 (at the time of diagnosis, he was 11 years old) came to the hospital with complaints of high-grade fever, fatigue, and bone pain. Upon admission to the hospital physically, some features of the hematological disorder, such as lymphadenopathy and splenomegaly, were revealed. The hematological analyzer showed an extremely high WBC count (429.78 × 10^9^/L), anemia (Hb 85 g/dL), and thrombocytopenia (PLT 47 × 10^9^/L). Morphological examination of the blood smear revealed 93.0% of blast cells in peripheral blood. Blood chemistry and blood coagulation parameters were normal. A morphological study of bone marrow aspirate detected 91% of blast cells. According to clinical guidelines, immunophenotyping using the flow cytometry method was performed. The following phenotype was established: CD10+ (100%), CD19+ (88%), CD20+ (63%), CD34+ (100%), CD45+ (65%), CD81+ (41%), CD24+ (22%), CyCD79a+ (72%), CD99+ (92%), CD79a+ (97%), CD22+ (18%). Fluorescence in situ hybridization (FISH) for BCR-ABL1 rearrangements was performed on the marrow specimen. The 50% Ph(+) cells were found.

After 15 days of induction therapy, the flow cytometry method did not determine minimal residual disease (MRD). After the course of treatment, the patient was discharged in remission. After a year since leukemia manifestation, no leukemic cells were observed in blood and bone marrow aspirate, and BCR-ABL fusion signal in bone marrow was not found by FISH as well.

**Fig. 1:**
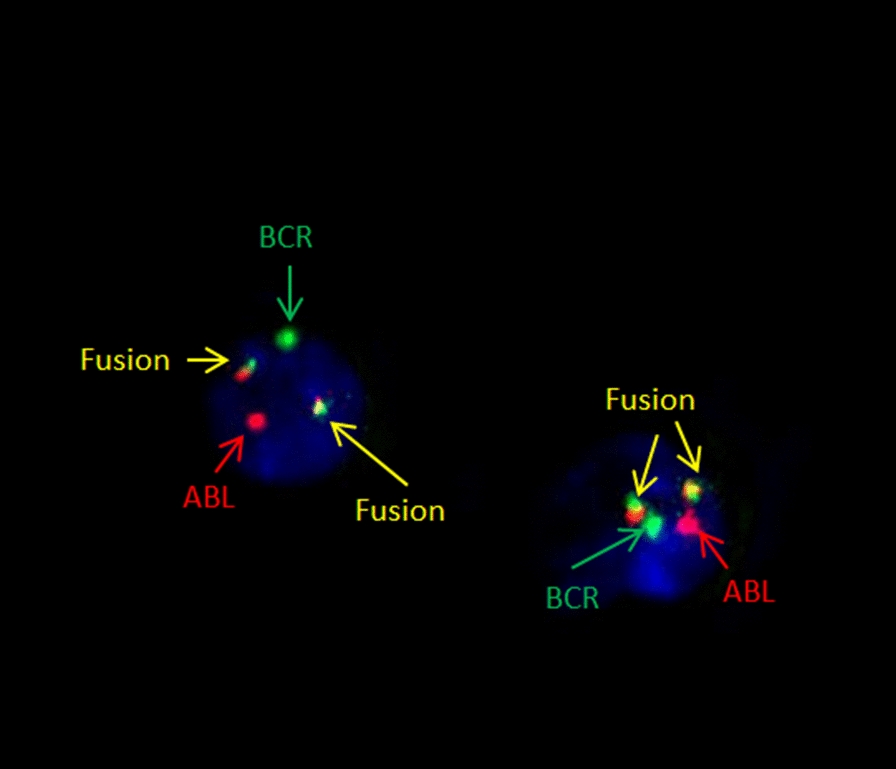
BCR/ABL1 dual fusion positive interphase nucleus in CSF; BCR/ABL1 and ABL/BCR positive metaphase cell (arrows indicate fusion products, BCR and ABL genes)

Four years after the initial diagnosis, the patient went to the doctor with neurological symptoms: complaints of headache, fatigue, strabismus, and diplopia. Symptoms of hematological disorders such as hepatosplenomegaly and lymphadenopathy were not found. Head MRI revealed nonspecific gliotic areas in cerebral white matter, idiopathic intracranial hypertension, and mild ventriculomegaly. The complete blood and bone marrow aspirate count data were within normal limits. FISH did not determine the BCR-ABL fusion signal in bone marrow and peripheral blood. The CSF was analyzed, and BCR-ABL1 rearrangements were performed on the specimen. The 90% Ph (+) cells were found in CSF (Fig. 1, Table 1).

**Table 1:** The results obtained from the FISH analysis

CSF sample/ number of the cells (cells/μl)	Total number of analyzed cells	Leukemic cells (%)	2 fusions + 1green + 1red	Normal cells (%)	2 green + 2 red
1.1300	200	90	180	10	20
2. 19	200	0	0	100	200

**Fig. 2:**
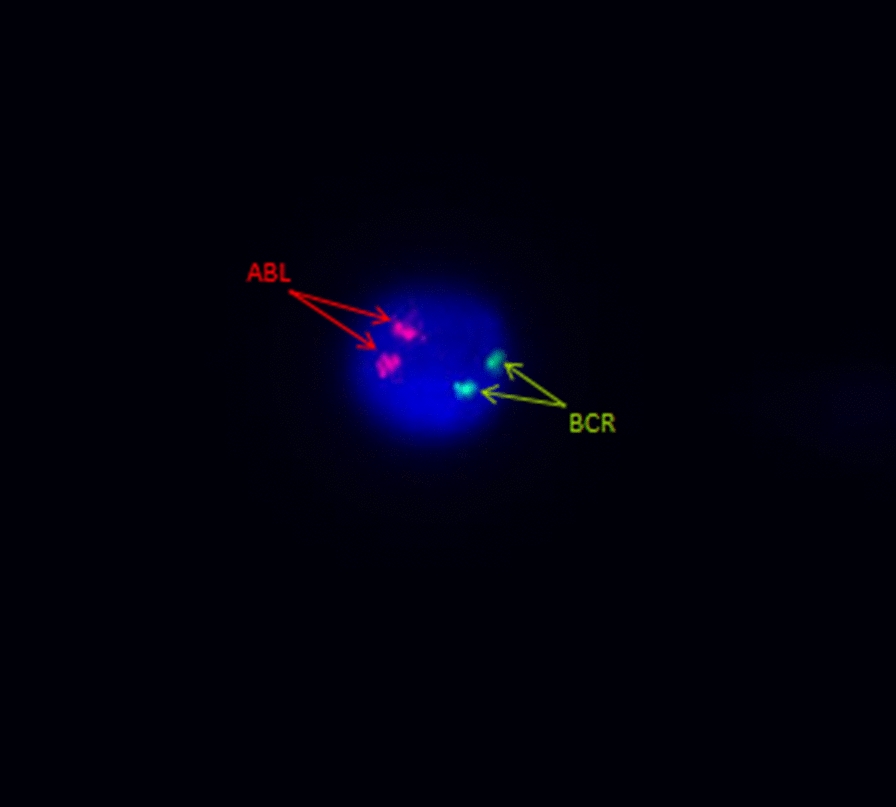
BCR/ABL1 negative interphase nucleus in CSF; (arrows indicate BCR and ABL genes)

To demonstrate the feasibility of our approach, we have included an additional sample with a lower rate of leukemic cells. The CSF white blood cell (WBC) count in this sample was 19 cells/μl. Despite the limited number of cells, we were able to successfully fix the cells, and our method performed flawlessly. In this particular case, we utilized the sample from a pediatric patient diagnosed with B-cell acute lymphoblastic leukemia (B ALL). Initial diagnostic tests did not reveal any abnormalities such as BCR-ABL translocation, ETV6-RUNX1 translocation, or MLL-rearrangements. However, the patient consistently exhibited high minimal residual disease (MRD) levels in the bone marrow, and the CSF was also affected. Using an in vitro diagnostic (IVD) BCR/ABL1 t(9;22) probe on the fixed CSF sample, the result was negative as expected (Fig. 2).

## Conclusion

The main difficulty in CSF analysis is the low cell amount in the sample. Though cytogenetic manuals suggest the iFISH cytospin protocol for CSF samples is cell fixation right on the slide [17], it is difficult to perform and requires practice. Here we suggest a greatly simplified standard iFISH method which is easy to implement and is not time-consuming; besides, mild processing of the CSF sample allows cells to remain undamaged. The described technique may be useful for iFISH analysis of sensitive low-cell samples. In this clinical case, we found that 90% of blast cells in the CSF were Ph-positive.

In conclusion, our proposed method might be a valuable tool for diagnosing and monitoring residual leukemic cells in CSF samples and could be a potential complementary test for detecting the presence of blast cells or residual leukemic cells in the case of ALL.

## Data Availability

Datasets for the current study are not publicly available to protect the anonymity of the respondents.
